# Decline in Work Ability over Time and Its Association with Physical Performance Among Senior Workers: A Prospective Cohort Study

**DOI:** 10.3390/ijerph23070842

**Published:** 2026-06-26

**Authors:** Simone Ejstrup, Niels-Peter Brøchner Nygaard, Gert Frank Thomsen, Inge Brosbøl Iversen, David Høyrup Christiansen, Bibi Gram

**Affiliations:** 1 Department of Physiotherapy and Occupational Therapy, Esbjerg and Grindsted Hospital, University Hospital of Southern Denmark, 6700 Esbjerg, Denmark; 2 Department of Regional Health Research, University of Southern Denmark, 5230 Odense, Denmark; niels-peter.brochner.nygaard@rsyd.dk (N.-P.B.N.); bgram@health.sdu.dk (B.G.); 3 Research Unit of Endocrinology, Bariatrics and Diabetes, Esbjerg and Grindsted Hospital, University Hospital of Southern Denmark, 6700 Esbjerg, Denmark; 4 Research Unit of Neurology, Esbjerg and Grindsted Hospital, University Hospital of Southern Denmark, 6700 Esbjerg, Denmark; 5 Department of Occupational and Environmental Medicine, Esbjerg and Grindsted Hospital, University Hospital of Southern Denmark, 7200 Grindsted, Denmarkinge.brosbol.iversen@rsyd.dk (I.B.I.); 6 Department of Clinical Medicine, Health, Aarhus University, 8000 Aarhus, Denmark; david.christiansen@midt.rm.dk; 7 University Clinic for Interdisciplinary Orthopedic Pathways, Elective Surgery Center, Silkeborg Regional Hospital, 8600 Silkeborg, Denmark

**Keywords:** aging, physical performance, physical work demand, work capacity

## Abstract

**Highlights:**

This prospective cohort study investigated the change in work ability among senior workers over a 6.5-year period. This paper presents additional cross-sectional analyses and combines longitudinal work ability data with objectively measured health outcomes.

**Public health relevance—How does this work relate to a public health issue?**

- There is an important need to investigate the change and predictors of work ability in the later stages of working life as the retirement age is increasing in many countries.
- Physical capacity is essential for meeting physical work demands and as it decreases with age, the aging workforce may be challenged.

**Public health significance—Why is this work of significance to public health?**

- Among senior workers, work ability decreases over time especially in the group of workers aged 56–59 years.
- Objectively measured physical performance shows that better performance is associated with higher scores of work ability.

**Public health implications—What are the key implications or messages for practitioners, policy makers and/or researchers in public health?**

- Promoting work ability among senior workers is essential for prolonging a healthy working life.

**Abstract:**

Background: Work ability (WA) decreases with age. This cohort study investigated changes in WA over time among senior workers and explored associations between WA and objectively measured physical performance. Methods: A questionnaire regarding WA (0–100 points), work status and general health was sent out to individuals aged 50–65 years (*n* = 23,463) with a 6.5-year follow-up. A subgroup participated in a clinical substudy testing physical performance. Results: A total of 13,404 participants responded to the baseline questionnaire (56%) and 8474 (63%) responded at follow-up. Overall, WA decreased by −8.3 points over time (95% CI: −8.9 to −7.7). The decline was similar between genders and among senior workers with high or low physical work demands. The steepest decline in WA was among those aged 56–59 years at baseline (−10.3 points, 95% CI: −11.5 to −9.1). Clinical examination showed that WA was positively associated with higher isokinetic muscle strength, handgrip strength and functional capacity after adjusting for confounders (all *p* < 0.05). Conclusions: Work ability decreased over time among all senior workers. Clinical subgroup analyses revealed positive associations between physical performance and WA. Future longitudinal studies are needed to determine whether improving physical performance can reduce age-related decline in work ability.

## 1. Introduction

Self-reported work ability (WA) is often used as an indicator of workers’ health, and previous prospective studies have established that WA tends to decline with age [1,2,3,4,5,6]. With retirement ages rising in many countries due to demographic changes and longer life expectancy [7,8,9], it is increasingly important to investigate changes and predictors of WA in the later stages of the working life. Age and physical capacity are recognized as important determinants of WA, and their association with WA have been documented in previous studies [1,2,3,4,5,6]. However, much of this evidence is derived from self-reported measures of physical activity, which may introduce bias and could potentially obscure the true physiological relationship.

Physical capacity is essential for meeting physical work demands and affects WA. Physical capacity is known to decline with increasing age, which can pose a challenge for older workers [10,11,12]. For example, lower limb muscle strength has been shown to decline by approximately 31% for males and 34% for females from the age of 25 to 75 [11]. Similarly, maximum oxygen consumption (VO_2_ max), decreases by approximately 10% per decade, amounting to a 58% reduction from ages 20 to 80 [11]. While physical work demands often remain unchanged, the reduced capacity can lead to work-related fatigue, work injuries, sickness absence or premature exit from the labor market, although individual variation and contextual factors should be considered [10,13]. Previous studies have emphasized the need for further research to identify potential factors that promote good WA among older workers [14,15,16]. Among these, physical factors such as muscle strength, cardiorespiratory fitness and functional capacity are particularly relevant, as loss of muscle strength and power are pronounced after the age of 60 [12]. To date, few studies have integrated objective performance-based measures of physical and functional capacity into a large cohort of senior workers. This represents a critical gap in understanding the precise mechanisms by which physical capacity preserves WA.

Individual trajectories for changes in WA may vary depending on occupational history, personal resources and physical and mental workload, e.g., WA among females decline more than males, physical work demands are associated with deeper decline in WA, and job control and relations at work influences the WA positively [4,5]. Workers in physically demanding jobs may experience a steeper decline in WA due to the mismatch between declining physical capacity and unchanged work demands [4]. It is important to estimate the changes in WA among different subgroups, such as gender and occupational groups, to better understand the mechanisms behind declining WA and to tailor targeted interventions. Understanding how WA changes over time and how it relates to physical and functional capacity may enhance future interventions to promote WA among senior workers.

### Objectives

The aim of this study was to investigate changes in WA over time among senior workers, with a focus on differences across gender, physical work demands and age groups. To complement these longitudinal analyses, a clinical substudy was conducted to examine the cross-sectional associations between WA and objectively measured physical and functional capacity, thereby providing insight into potential underlying mechanisms of observed changes in WA.

For the cohort study, we hypothesized that WA would decline over time and that the decline would be greater among women, workers with high physical work demands and among the oldest group of workers, since we expected that these three groups would have lower physical performance and thus decreased WA. For the clinical substudy, we expected that better physical and functional capacity would be associated with better WA.

## 2. Materials and Methods

### 2.1. Survey Cohort

The study is based on a population-based cohort [17]. Individuals born between 1952 and 1966 who lived in Esbjerg municipality, Denmark, in December 2016 (*n* = 23,463) were invited to participate. Names and social security numbers were obtained from the Danish Health Data Authority. All participants received a comprehensive questionnaire including items regarding work, WA, health status, musculoskeletal disorders, and ergonomic exposures. The questionnaire was sent out via electronic mailbox (e-Boks) or by conventional mail. The baseline questionnaire was answered individually between October 2017 and November 2020 (13,599 responders). The follow-up data was collected between December 2024 and October 2025 (8474 responders).

### 2.2. Clinical Substudy

Between baseline and follow-up, a clinical subgroup was formed from the survey cohort. Since we were not able to find a minimal clinically important difference (MCID) of WA in healthy subjects, our power calculation was based on a previous study with a 0.75 mean WA difference between blue- and white-collar workers in work ability score (0–10 points, 7.5 points on a 0–100 scale), and a standard deviation (SD) at 1.7 [18]. With a power at 0.8 and alpha at 0.05, a sample of 252 participants were required to secure a representative sample from the survey cohort. Participants from the survey cohort, who had regular employment when answering the baseline questionnaire (*n* = 9263) were stratified into twelve groups based on gender, age groups (55–59 years, 60–64 years and 65–70 years) and occupational group (blue- and white-collar workers) to achieve sufficient distribution of age, gender, and occupational groups. The participants were stratified with approximately 28 participants in 12 strata (*n* = 336) (Appendix A). To minimize selection bias, participants were randomized to determine the order in which they were invited to the clinical substudy. Randomization was conducted separately within each stratum and based on the date of completion the baseline questionnaire to achieve a similar interval between baseline and clinical testing (five years ± 6 months). The invitations were sent out on a rolling basis and continued until each stratum reached the target sample (*n* = 28) or no further eligible participants remained. The eligibility criteria for participating in the clinical substudy were (I) could read and talk Danish, (II) able to perform the physical test battery without any medical or physical issues, e.g., amputation or heart failure.

### 2.3. Ethics

The study has been approved by The Regional Committees on Health Research Ethics for Southern Denmark (project ID: S-20180162) and was conducted in accordance with the declaration of Helsinki. A panel of patients and relatives at Esbjerg Hospital, University Hospital of Southern Denmark, discussed and approved the content and setup for the study. For the clinical substudy, the participants received both written and oral information about the study and provided their written informed consent before participating in the clinical examination.

The study follows the guidelines for the reporting of observational studies in epidemiology (STROBE) [19]. During the preparation of this manuscript, the first author used Microsoft Copilot (Microsoft, Redmond, WA, USA; M365 Copilot, GPT-5 chat model) for the purposes of helping with coding in Stata statistical software, version 19.0 (StataCorp LLC., College Station, TX, USA) and superficial text editions. The authors have reviewed and edited the output and take full responsibility for the content of this publication.

### 2.4. Outcomes

#### 2.4.1. Survey Cohort

##### Work Ability Score

The work ability score (WAS) was measured with a single item on a visual analog scale (VAS) from 0 to 100, where zero means ‘not able to work’ and 100 means ‘best imaginable work ability’. Since previous research has established a strong correlation between WAS and the full Work Ability Index (Spearman correlation coefficient = 0.63, *p* < 0.001) [20], the single item WAS was used. In the clinical substudy, the WAS from 0 to 100 had a strong correlation with the original work ability 0–10 scale [21,22] (Pearson’s correlation coefficient = 0.8361, *p* < 0.001).

For practical interpretation, WAS was categorized as poor (0–50 points), moderate (51–70 points), good (71–90 points) and excellent (91–100 points), in relation to Ilmarinen’s work ability score (0–10) [20,23]. The analysis uses WAS as a continuous variable, but in the categorizing is also presented in the characteristics of the participants.

##### Physical Work Demands

The physical work demands were based on self-reported estimates of ergonomic exposures. The questions were: ‘How much of your working time do you …’ (I) walk or stand?; (II) work with twisted or bend back without support from the hand and the arms?; (III) have the arms lifted to or above shoulder height?; (IV) do the same arm movements several times a minute (e.g., package work, mounting, machine feeding, carving)?; (V) squat or kneel when you work?; (VI) push or pull?; and (VII) lift or carry? The response options for each of the seven questions were: almost all the time (100 points), approximately 3/4 of the time (75 points), approximately 1/2 of the time (50 points), approximately 1/4 of the time (25 points), seldom/very little (12.5 points) and never (0 points). The seven variables were averaged to give a combined ergonomic index of 0–100. Finally, the participants were categorized into high physical work demands (≥20 points) or low physical work demands (<20 points). The low cut-point was chosen since a majority of the participants with low physical work demands were found below 20 points in the ergonomic index, similar Andersen et al. [13].

##### Leisure-Time Physical Activity

The participants were asked to describe their leisure-time physical activity (LTPA), where walking and cycling as transport to and from work and grocery shopping should be included. LTPA were assessed with three statements: (I) ‘Walking, cycling or other light exercise where you do not get out of breath or sweat (e.g., Sunday walks, light gardening)’; (II) ‘recreational sports, heavy gardening or fast walking/cycling where you sweat and get out of breath’; and (III) ‘High intensity training or competitive sports’. The response categories were: ‘Does not perform the activity’, ‘Under two hours per week’, ‘Two to four hours per week’ and ‘More than four hours per week’. Afterwards the variable was dichotomized into ‘moderate to high LTPA’ and ‘none to light LTPA’. Participants reporting above two hours in statement II and III were coded as ‘moderate to high LTPA’, all responses in statement I and less than two hours in statement II and III were coded as ‘none to light LTPA’. The questions are adopted from the questionnaire ‘Work environment and health 2018’, conducted by The National Research Centre for the Working Environment (NFA) [24].

#### 2.4.2. Clinical Substudy

The clinical substudy was performed in test-lab facilities at Esbjerg Hospital, University Hospital of Southern Denmark. In order to secure consistency in test procedures, the intention was to minimize the number of testers and use a thoroughly prepared protocol regarding tests and instructions.

##### Muscle Strength

The isokinetic and isometric muscle strength was tested for the shoulder and the knee using a Biodex System 4 Pro dynamometer^®^ (Biodex Medical Systems, Shirley, NY, USA). The dominant arm and the non-dominant leg were tested. The isokinetic tests included three maximal concentric and eccentric contractions separated by 15 s’ rest. The isometric tests included three maximal contractions lasting 3–5 s separated by 40 s’ rest. The anatomical axis of rotation was aligned to the dynamometer axis using visual inspection and manual palpation as recommended from Biodex and Harbo et al. [25]. A break of three minutes separated each test. Muscle tests in the shoulder were performed testing the abduction and adduction. Range of motion (ROM) in the isokinetic test was set to 20–90 degrees abduction in the shoulder, and the isometric test was performed in 45 degrees abduction. For the muscle strength in the knee, flexion and extension were tested. ROM was set to 10–90 degrees flexion for the isokinetic test, and the isometric test was tested in 90 degrees flexion in the knee. The maximum peak torque for each test was used for further analysis.

Additionally, isometric handgrip strength (HGS) in the dominant hand was tested using a handheld dynamometer (Jamar Plus, Patterson Medical, Warrenville, IL, USA) in handgrip position two (hand size position) for all participants [26]. The participants performed three maximal muscle contractions lasting 3–5 s separated by 15 s’ rest. The highest attempt was used to determine maximal handgrip strength.

##### Cardiorespiratory Fitness

The estimated maximal oxygen uptake (VO_2_-max) was assessed on an ergometer bike using the submaximal Astrand test [27]. The workload started at 450 kilopond meters/min (73.5 Watts) and the participants were instructed to keep the cadence around 60 revolutions per minute (RPM) throughout the test. For each individual, the workload was manually and gradually increased during the first three minutes to reach the heart rate limit between 118 and 170 beats per minute (bpm). The VO_2_-max was estimated by Astrands nomogram as a result of workload, steady state heart rate, sex, age and weight in kg [28].

##### Functional Testing

The physical function of the participants was assessed by the following three tests: 30 s sit-to-stand test (30STS), single leg stance test (SLST) and stairclimbing test (SCT).

The 30STS is used as a proxy measure of lower limb muscle strength, balance control and functional abilities primarily in the oldest population [29]. For 30STS, the participants were placed in an armless chair (seat height 46 cm, seat depth 42 cm) and instructed to complete as many full stands as possible in 30 s. The participants should place their arms across their chest and were not allowed to use them during the test. They had one attempt and the number of full stands were noted [29].

Performing the SLST, the participants were instructed to stand on one leg for a maximum of 60 s. Their hands should be placed on the hips, and the legs were not allowed to touch each other. If the participant moved their standing foot, moved their hands away from their hips or lost their balance, the timing stopped. The best attempt out of the three was noted [30].

SCT assessed the participants’ lower limb muscle power and balance. The participants were instructed to ascend and descend a stair with nine steps as fast but safe as possible (step height: 17 cm, step depth 28.5 cm). They had one attempt, and the time was noted in seconds (faster performance = lower seconds) [31].

##### Leisure-Time Physical Activity

In the clinical substudy, LTPA was assessed with the International Physical Activity Questionnaire Long Form (IPAQ) by domains ‘the leisure time’, ‘active transportation’ and ‘domestic and garden’, consisting of walking, cycling, moderate-intensity activities and vigorous-intensity activities [32,33]. The outcome was reported as metabolic equivalent minutes per week (MET-min/week) and the total weekly hours of LTPA. Data were cleaned according to the IPAQ guideline [34].

#### 2.4.3. Covariates

The following covariates were collected from the baseline questionnaire: (I) age in years; (II) gender (male or female); (III) employment status (regular employment, retired, early retirement, flex job (subsidized employment for people with disabilities), unemployed, on social security, economic self-supported or other); (IV) self-reported working hours on an average week; (V) smoking status (yes, non-smoker, former smoker); (VI) number of chronic diseases (asthma, diabetes, cardiovascular diseases, cancer and mental diseases); and (VII) body mass index (BMI, kg/m^2^) calculated by self-reported height and weight. In clinical substudy, BMI was calculated by objectively measured body weight and height. Age, working hours and BMI were handled as continuous variables whereas gender, employment status, smoking status and number of chronic diseases were handled as categorical variables in the analyses.

### 2.5. Statistical Methods

#### 2.5.1. Survey Cohort

Data were described using group mean values and SD for continuous variables. Categorical variables were described as frequency and percentages. Regarding change in WAS over time, a mixed linear regression model was used using restricted maximum likelihood estimation (REML). A random intercept for each participant was included to account for within-subject correlation due to repeated measures, while all confounders, time and interaction terms, were modeled as fixed effects. Given the two time points per participant, random slopes were not included. Data were reported with a predicted mean and 95% confidence intervals (95% CI) for baseline and follow-up and change in WAS over time. Furthermore, mixed linear regression model was conducted to investigate the interaction between time and gender, time and physical work demands, and time and age groups (≤55 years, 56–59 years and ≥60 years at baseline). In addition, the model was adjusted for the baseline variables BMI, number of chronic diseases, smoking status, LTPA and working status (dichotomized to workers and non-workers). Due to the relatively long period for baseline data collection (2017–2020), a likelihood ratio test based on maximum likelihood estimation was conducted to see if adding answering year as a confounder improved the model fit. It did not improve the model fit (*p* = 0.254) and was therefore not included in the final models. A *p*-value ≤ 0.05 was considered statistically significant.

Furthermore, we conducted an explorative analysis driven by the data. From a clinical perspective this was done to focus on the participants with the poorest WA, by investigating the differences in baseline characteristics between participants reporting poor WA (WAS < 51 points) and participants reporting good, moderate and excellent WA (WAS ≥ 51 points) combined as a new category named high WA (*t*-test for continuous variables and chi^2^ test for categorical variables). Missing data in the outcome variable were handled using likelihood-based estimation within the mixed model which allows inclusion of participants with incomplete WA data [35]. In contrast, observations with missing values in covariates were excluded from the analyses (complete-case approach). Appendix B presents frequencies and percentages of the missing data for each variable.

#### 2.5.2. Clinical Substudy

Multiple linear regressions were used to estimate associations between WAS measured at the visit for the clinical examination and exposures (muscle strength, estimated VO_2_-max and functional capacity). Data were reported with a mean (SD) and a regression coefficient with 95% CI. The analyses were adjusted for gender, age, physical work demands, working status (dichotomized to workers and non-workers), BMI, smoking status, LTPA and number of chronic diseases since these eight variables are considered confounders. Potential confounders were based on a priori knowledge from previous studies [16,17,36,37]. All analyses did not violate the model assumptions regarding linearity. Multicollinearity was assed using variance inflation factors (VIF), and for all analyses the factors were below five (range: 1.66–2.12) indicating moderate but acceptable multicollinearity [38]. All analyses were performed using Stata statistical software, version 19.0 (StataCorp LLC., College Station, TX, USA).

## 3. Results

### 3.1. Survey Cohort

**Figure 1:**
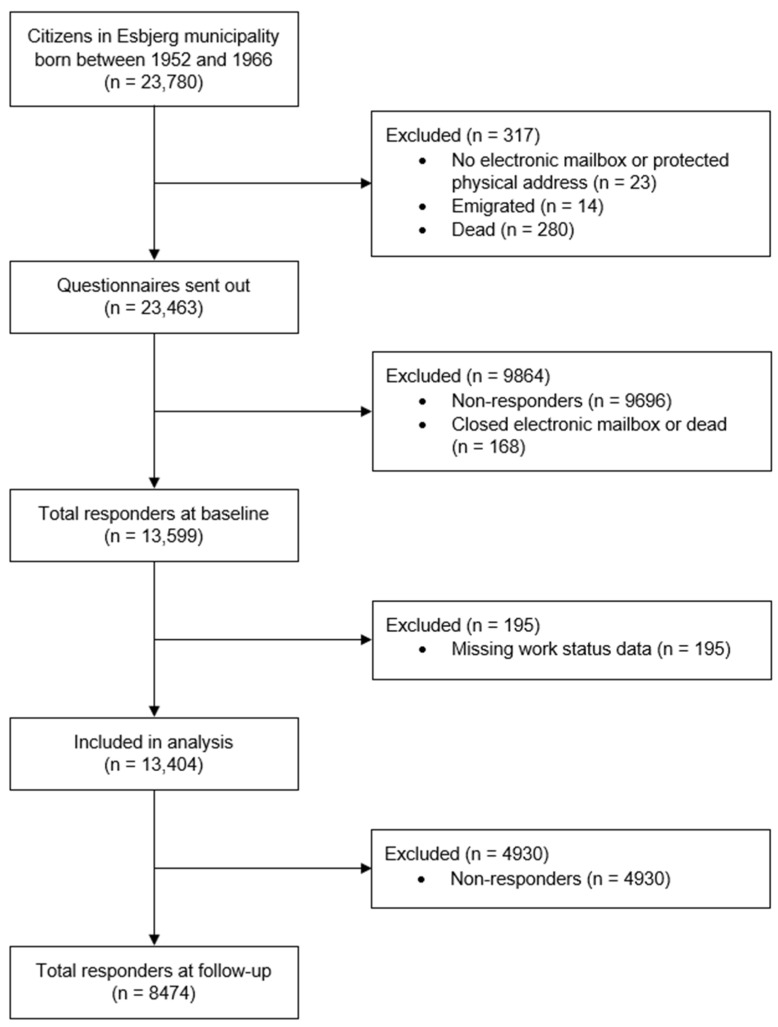
Flowchart of responses to the questionnaires. [*n* = numbers].

In December 2016, the identities of 23,780 persons were acquired from Statistics Denmark. Among these, 317 were excluded from participation due to emigration (*n* = 14), death (*n* = 280), no electronic mailbox or protected physical address (*n* = 23). Of the 13,599 (57%) participants who responded at baseline, 195 were excluded due to non-respondence to the question regarding working status, leaving 13,404 for analysis. At follow-up 6.5 years later, 8474 of the 13,404 participants (63%) responded to the questionnaire (Figure 1).

### 3.2. Clinical Substudy

Out of the 13,599 baseline responders, 9263 (69%) were regularly employed. From this group, 1951 were randomly selected as eligible for the clinical study. Between November 2022 and April 2024, 436 participants responded to the invitation. Among these, 331 participants were included in the clinical substudy (targeted sample size = 336). Participants were excluded if there were already enough participants in the specific stratum (*n* = 99), were too sick to participate or had an injury (*n* = 3) or did not want to participate (*n* = 3). In total, eleven of the twelve strata reached their target sample size (Appendix A).

### 3.3. Descpritive Data

The survey cohort consisted of 13,404 participants (mean age 59 years, SD 4.2) with 53% females at baseline and WAS at 75.6 points (SD 28.5) (Table 1). At follow-up 6.5 years later, 8474 participants had answered the questionnaire (mean age 65.6 years, SD 4.3) with 52% females. They reported WAS at 70.2 points (SD 29.7). Overall, several participants had left the labor market and those who worked did so fewer hours per week. Participants lost to follow-up had significantly lower baseline WA, were less often in regular employment, held more frequent jobs with high physical work demands and were unhealthier (Appendix C). Missing values differed from 8% (number of chronic diseases at baseline) to 56% (self-reported physical work demands at follow-up) (Appendix B).

Among the 331 participants in the clinical substudy (mean age 62.1 years, SD 4.0) 49% were females and 74% in a regular employment (Table 1). On average they scored 78.8 points (SD 22.9) on WAS at the time of their clinical test. Additionally, participants in the clinical substudy were younger, more often in regular employment, healthier and reported higher WAS when compared to rest of the cohort at baseline (Appendix D).

### 3.4. Change in WA in the Survey Cohort

**Figure 2:**
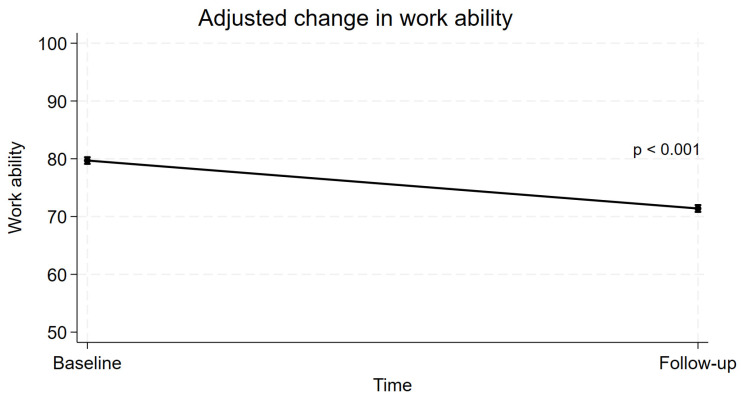
The adjusted change in work ability: −8.3 points (95% CI: −8.9 to −7.7) (adjusted for working status (workers vs. non-workers), body mass index, smoking status, number of chronic diseases and leisure-time physical activity). The adjusted analysis included also interactions between time and gender, time and physical work demands, and time and age groups (≤55 years, 56–59 years and ≥60 years at baseline).

**Figure 3:**
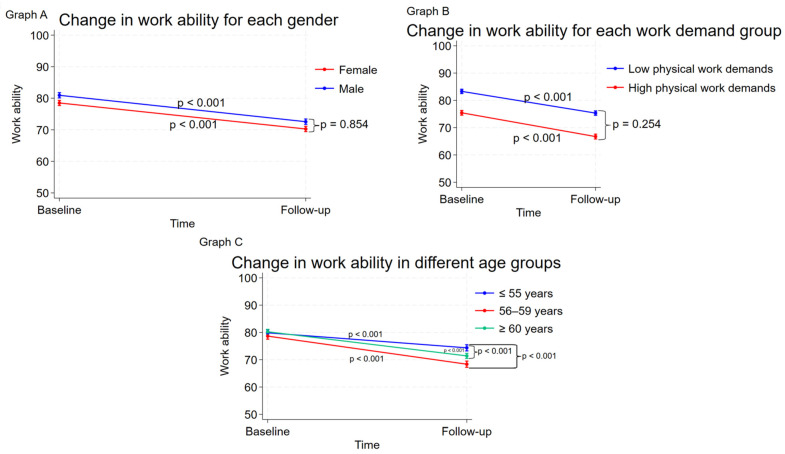
Change in work ability among different subgroups. Graph (**A**) is the differences between males (blue line) (change: −8.4 points, 95% CI: −9.3 to −7.5) and females (red line) (change: −8.2 points, 95% CI: −9.1 to −7.3). Graph (**B**) is the difference between low physical work demands (blue line) (change: −8.0 points, 95% CI: −8.8 to −7.1) and high physical work demands (red line) (change: −8.7 points, 95% CI: −9.7 to –7.8). Graph (**C**) is the differences between the three age groups, ≤55 years (blue line, reference group) (change: −5.5, 95% CI: −6.7 to −4.3), 56–59 years (red line) (change: −10.3 points, 95% CI: −11.5 to −9.1) and ≥60 years (green line) (change: −8.8, 95% CI: −9.8 to −7.9).

Overall, there was a decline in WA between baseline and follow-up (crude: −8.0 points, 95% CI: −8.7 to −7.4, adjusted: −8.3 points, 95% CI: −8.9 to −7.7) (Figure 2). The decline in WA was not statistically different between males and females (*p* = 0.854) or between senior workers with low physical work demands and senior workers with high physical work demands (*p* = 0.254); whereas the slopes differed significantly between age groups (*p* < 0.001) (Figure 3A–C). Males, females, workers with high physical work demands and workers with low physical work demands had decline in WA between −8.0 to −8.7 points after adjusting for BMI, working status, smoking status, number of chronic diseases and LTPA (combined 95% CI: −9.7 to −7.1) (Figure 3A,B). The decline was most pronounced among participants aged 56–59 years, with –10.3 points change (95% CI: −11.5 to −9.1) (Figure 3C).

### 3.5. Explorative Analysis for the Survey Cohort

The participants reporting poor WA at baseline differed from the rest of the cohort across all variables and reported poorer health (Table 2). They were more likely to have high physical work demands (67% vs. 47%), had slightly higher BMI (28.0 kg/m^2^ vs. 26.9 kg/m^2^), and higher representation of smokers (27% vs. 16%). In addition, fewer reported no chronic diseases (42% vs. 72%) and even fewer reported moderate to high LTPA (29% vs. 44%). All comparisons were statistically significant (*p* < 0.001).

At follow-up, 55% of the participants who reported poor WA at baseline (*n* = 1034), responded to the questionnaire, 76% were retired and 41% completed the WAS. The WA increased over time among senior workers reporting poor WA at baseline. In the crude model, the WA increased with 14.0 points (95% CI: 11.8 to 15.4, *p* < 0.001). In the adjusted model, WA increased with 13.8 points (95% CI: 11.9 to 16.0, *p* < 0.001), when adjusting for BMI, smoking status, number of chronic diseases, LTPA and working status, including interactions between time and gender, time and physical work demands, and time and age groups.

### 3.6. Clinical Substudy Analysis

All unadjusted associations between WAS and physical and functional capacity revealed statistically significant results, indicating better physical performance is associated with higher WAS (Table 3). The associations between WAS and the isokinetic knee flexion (KF) and shoulder abduction (SA) and HGS revealed positive statistically significant associations when adjusting for age, gender, physical work demands, working status, BMI, smoking status, number of chronic diseases and LTPA. The association between cardiorespiratory fitness and WA was not statistically significant in the adjusted model. 

Lastly, all three functional tests were positively associated with WAS; more repetitions in sit-to-stand, longer time standing on one leg and faster stairclimbing were associated with higher WAS (Table 3).

## 4. Discussion

The aim of this prospective cohort study was to investigate changes in WA over time among senior workers. Overall, WA decreased over a 6.5-year period. Female workers and senior workers with high physical work demands consistently reported lower WA than males and workers with low physical work demands, but the declines over time were similar. The steepest decline occurred among senior workers aged 56–59 years. The clinical substudy further demonstrated positive associations between physical performance and health.

These findings align with previous longitudinal studies showing age-related declines in WA [1,2,3,4,5]. Since only one study reported WA as a continuous variable [3], direct comparisons are limited. In the present study, the decline in WAS was reflected in a significant shift in the distribution of WA categories. The proportion of senior workers reporting poor work ability (<51 points) increased from 18% at baseline to 26% at follow-up, indicating that the mean decline pushed a substantial segment of the cohort into a critical range. The result underlines that focus on work ability among senior workers is relevant and initiatives promoting WA should be prioritized among this group of workers. Explorative analyses showed that participants reporting poor WA at baseline were unhealthier and the clinical subgroup analysis showed positive associations between WA and physical performance. In addition, the explorative analyses showed that participants reporting poor WA at baseline were more often out of the labor market, which is consistent with previous studies reporting association between low WA and premature workforce exit [37,39,40,41]. This highlights the importance of focusing on workers reporting poor WA in interventions and clinical setting, since maintaining high WA increases the likelihood of remaining in the workforce [42].

Contrary to our hypothesis, the steepest decline in WA was not observed in the oldest group but among workers aged 56–59 years. An explanation, could be that those who worked above 60 years represented a more robust subset of workers, whereby participants above 60 years reporting poor WA may exit the labor market earlier [37].

Another surprising finding was the similar decline between genders and physical work demands. Although female workers and workers with high physical work demands generally report lower WA compared to their counterparts, the decline over time was similar in present study. These findings add nuance to the existing literature [4,5], suggesting that the relationship between gender, physical work demands and decline in WA may be more complex and depend on context.

The unexpected increase in WA among senior workers reporting poor WA should be interpreted cautiously. This pattern likely reflects regression to the mean and selection and information bias [43]. A healthy worker effect is likely inflating follow-up WAS, as non-responders at follow-up tended to be unhealthier, less often regular employees and poor work ability at baseline (Appendix C). This may have resulted in a relatively healthier subpopulation at follow-up. Additionally, recall bias may have occurred because some participants were no longer employed and may have underestimated former work demands, and thereby overestimating their WA.

Findings from the clinical substudy indicate that better physical performance is associated with higher WA among senior workers, aligning with previously cross-sectional studies of older workers [44,45] and a systematic review [46]. The present study did not find statistically significant association between cardiorespiratory fitness and WA. This contradicts previous studies, indicating a positive association between these two [16,45], but the test methods differ and could be an explanation for different results.

Although a MCID has not been established for WA in healthy population, this present study demonstrated potentially relevant association between WA and isokinetic strength, HGS and functional capacity, as an increase of 1 Nm/kg was associated with 1–5-point improvement in WA. Compared with reference values, participants demonstrated normal muscle strength [25,47], cardiorespiratory fitness [48] and functional capacity [47], although mean values were at the lowest end of the normal range.

The number of confounders is high relative to the clinical sample size; it may raise concerns about overfitting. But the similarity between crude and adjusted coefficients along with comparable small CI, suggests that model overfitting is unlikely to have substantially influenced the results. Furthermore, covariates were selected a priori based on previous studies and theoretical relevance. Aging is associated with declining physical capacity [11], and present study showed that higher physical performance was associated with better work ability. These findings highlight the importance of physical performance for sustaining WA among senior workers. As physical capacity declines, the ability to meet physical job demands may be compromised, potentially leading to a mismatch between physical capacity and work demands, resulting in reduced work ability. Longitudinal studies are needed to examine how these changes affect the balance between physical capacity and work demands over time.

### 4.1. Strength and Limitations

A strength of this study is its longitudinal design, allowing investigation of changes in WA over time. Another strength is the additional clinical substudy, which examined associations between objectively measured physical factors and WA. Over 85% of the data collection was performed by the same experienced physiotherapist, minimizing intertester variations. The predefined protocol ensured consistency and reliability in the test procedures.

This study has limitations that potentially could have affected the results. These limitations include possibility of recall bias, selection bias, healthy worker effect and design limitations. Recall bias could have introduced misclassification, as participants may not accurately remember the exact level of their exposures. Selection bias is possible, represented in high frequencies of missing values in some variables (e.g., 56% in self-reported physical work demands at follow-up), unhealthier non-responders at follow-up and non-responders at baseline. Previous research has shown that non-responders in cohort studies often differ systematically from responders by being unhealthier and less favorable socioeconomic characteristics [49]. Another explanation for non-respondence could be difficulties with completing the questionnaire because of cognitive deficits. In our study, the significant differences between responders and non-responders may have underestimated the associations.

Although the study included both workers and non-workers to limit the healthy worker effect, a residual selection bias has likely remained in the clinical substudy, which excluded participants who were too fragile to perform physical tests for 2–3 h. Sensitivity analyses revealed that workers reported significantly higher WAS, were younger, had higher muscle strength in some tests, cardiorespiratory fitness and better physical function compared to non-workers (Appendix E). This supports the presence of the healthy worker effect and could potentially obscure the true association between physical performance and WA. Additionally, only 4% of participants in the clinical substudy reported poor work ability in the baseline questionnaire and were healthier in several variables compared to the rest of the cohort, indicating selection bias (Appendix D).

Finally, there are a few limitations regarding the design of the study. The assessment of LTPA differed between the survey cohort and the clinical subgroup which affects the comparability across analyses. Additionally, self-reported LTPA may have introduced an information bias and measurements such as accelerometers would possibly give more objective estimation on LTPA. Lastly, the cross-sectional design limits causal interpretations.

### 4.2. Interpretation and Generalizability

This study is based on a representative sample since Esbjerg municipality encompasses both urban and rural areas, strengthening the external validity since Esbjerg is the fifth largest city in Denmark, although caution must be taken when generalizing to other countries. Focusing on the oldest group of workers is essential as changes in WA during the final years of working life are insufficiently explored. However, caution must be taken when generalizing the results, since loss to follow-up indicated that responders tend to be healthier than non-responders.

A decrease in eight points in WAS may be considered relevant and is consistent with the assumptions underlying the power calculation, which specified a difference of 7.5 points. From a clinical perspective, categorization of WA may be more informative, as a shift to a lower category reflects a meaningful decline and may indicate a need for workplace or clinical interventions.

## 5. Conclusions

Work ability declined over time among senior workers, with the most pronounced decline observed among senior workers aged 56–59 years. Female workers and senior workers with high physical work demands reported consistently lower work ability compared to their counterparts, but the decline was similar across subgroups. These findings were complemented by our objective clinical assessments, which revealed a positive cross-sectional association between higher muscle strength, better functional capacity and better work ability. Future longitudinal studies are needed to determine whether improving physical performance can reduce age-related declines in work ability.

## Figures and Tables

**Table 1:** Characteristics of the survey cohort and the clinical substudy. [*n* = numbers; SD = standard deviation; BMI = body mass index; LTPA = leisure-time physical activity; IQR = Interquartile range; NA = not available].

Characteristics	Survey Cohort		Clinical Substudy
	Baseline (*n* = 13,404)	Follow-Up (*n* = 8474)	(*n* = 331)
Age (years), mean (SD) Minimum–maximum	59.0 (4.2) 50.9–68.0	65.6 (4.3) 57.9–73.0	62.1 (4.0) 55–71
Gender, *n* (%) Male Female	6355 (47%) 7049 (53%)	4036 (48%) 4438 (52%)	168 (51%) 163 (49%)
Employment status, *n* (%) Regular employment Retired Early retirement Partly subsidized work Unemployed On social security Economic self-supported Other	9263 (69%) 1930 (14%) 1053 (8%) 575 (4%) 306 (2%) 164 (1%) 77 (1%) 36 (1%)	3786 (45%) 3819 (45%) 323 (4%) 220 (2%) 84 (1%) 10 (0%) 0 (0%) 232 (3%)	244 (74%) 59 (18%) 20 (6%) 1 (0%) 4 (1%) 0 (0%) 3 (1%) 0 (0%)
Work ability score (0–100 points), mean (SD) Excellent work ability, *n* (%) Good work ability, *n* (%) Moderate work ability, *n* (%) Poor work ability, *n* (%)	75.6 (28.5) 3945 (37%) 3846 (37%) 859 (8%) 1884 (18%)	70.2 (29.7) 1921 (29%) 2480 (37%) 561 (8%) 1786 (26%)	78.8 (22.9) 105 (32%) 157 (47%) 28 (9%) 41 (12%)
Average weekly working hours, mean (SD)	37.7 (10.7)	34.9 (11.6)	32.5 (14.0)
Self-reported physical work demands, *n* (%) High physical work demands Low physical work demands	6090 (51%) 5959 (49%)	1552 (41%) 2191 (59%)	154 (47%) 177 (53%)
BMI (kg/m^2^), mean (SD)	27.0 (5.0)	27.0 (4.9)	27.3 (4.8)
Smoking status, *n* (%) Yes Former smoker Non-smoker	2278 (19%) 2580 (21%) 7284 (60%)	903 (12%) 2205 (29%) 4537 (59%)	29 (9%) 130 (39%) 172 (52%)
Chronic diseases, *n* (%) 0 diseases 1 disease 2 diseases 3–4 diseases 5 diseases	8086 (66%) 3312 (27%) 778 (6%) 116 (1%) 1 (0%)	4277 (55%) 2620 (34%) 724 (9%) 136 (2%) 1 (0%)	177 (54%) 126 (38%) 24 (7%) 4 (1%) 0 (0%)
Leisure-time physical activity, *n* (%) None to light LTPA Moderate to high LTPA METS-min/week, median [IQR] Hours/week, median [IQR]	7161 (59%) 4952 (41%) NA NA	4359 (57%) 3286 (43%) NA NA	4014 [2481–6415] 16.4 [10.0–26.5]

Information regarding missing data is provided in Appendix B.

**Table 2:** Differences in baseline characteristics for participants reporting high work ability (≥51 points) and participants reporting poor work ability (<51 points). [High work ability = participants reporting excellent, good or moderate work ability combined; *n* = numbers; SD = standard deviation; BMI = body mass index; LTPA = leisure-time physical activity].

Characteristics	Baseline High Work Ability (*n* = 8650)	Baseline Poor Work Ability (*n* = 1884)	*p*-Value *
Age (years), mean (SD) Minimum–maximum	58.7 (4.2) 50.9–68.0	60.0 (4.2) 50.8–68.0	**<0.001**
Gender, *n* (%) Male Female	4265 (49%) 4385 (51%)	768 (41%) 1116 (59%)	**<0.001**
Employment status, *n* (%) Regular employment Retired Early retirement Partly subsidized work Unemployed On social security Economic self-supported Other	7188 (83%) 483 (6%) 610 (7%) 121 (1%) 169 (2%) 26 (0%) 40 (1%) 13 (0%)	384 (20%) 869 (46%) 173 (9%) 280 (15%) 53 (3%) 96 (5%) 18 (1%) 11 (1%)	**<0.001**
Work ability score (0–100 points), mean (SD) Minimum–maximum	87.5 (11.7) 51–100	21.0 (16.7) 0–50	**<0.001**
Average weekly working hours, mean (SD)	38.8 (9.5)	26.1 (15.1)	**<0.001**
Self-reported physical work demands, *n* (%) High physical work demands Low physical work demands	3997 (47%) 4565 (53%)	1018 (67%) 497 (33%)	**<0.001**
BMI (kg/m^2^), mean (SD)	26.9 (4.7)	28.0 (5.8)	**<0.001**
Smoking status, *n* (%) Yes Previously smoker Non-smoker	1395 (16%) 1817 (21%) 5414 (63%)	501 (27%) 440 (23%) 936 (50%)	**<0.001**
Chronic diseases, *n* (%) 0 diseases 1 disease 2 diseases 3–4 diseases 5 diseases	6190 (72%) 2069 (24%) 363 (4%) 28 (0%) 0 (0%)	786 (42%) 750 (40%) 281 (15%) 62 (3%) 1 (0%)	**<0.001**
Leisure-time physical activity, *n* (%) None to light LTPA Moderate to high LTPA	4860 (56%) 3762 (44%)	1331 (71%) 531 (29%)	**<0.001**

* = *T*-test for continuous variables and chi^2^ test for categorical variables. Statistically significant results (*p* < 0.005) are marked in bold. Information regarding missing data is provided in Appendix B.

**Table 3:** Associations between work ability score (outcome) and physical and functional exposures within the clinical substudy. [SD = standard deviation; *n* = numbers; WAS = work ability score (0–100 points); CI = confidence interval; Nm = newton meter; kg = kilograms; mL = milliliters; min = minutes; 30STS = 30 s sit-to-stand test; n = repetitions; SLST = single leg stance test; s = seconds; SCT = stairclimbing test].

Exposures	Mean (SD) (*n* = 331)	Crude Regression Coefficient for WAS (95% CI)	Adjusted Regression Coefficient for WAS (95% CI)
Knee (Nm) Isometric extension Isokinetic extension Isokinetic flexion	166.3 (58.6) ^1^ 129.8 (37.8) 66.1 (21.3)	**0.1 (0.1 to 0.1)** **0.1 (0.1 to 0.2)** **0.3 (0.2 to 0.4)**	0.1 (−0.01 to 0.1) 0.1 (−0.02 to 0.2) **0.2 (0.1 to 0.4)**
Shoulder (Nm) Isometric abduction Isokinetic abduction Isokinetic adduction	52.3 (18.0) ^1^ 46.8 (15.4) 67.7 (25.6)	**0.3 (0.1 to 0.4)** **0.3 (0.2 to 0.5)** **0.2 (0.1 to 0.3)**	0.2 (−0.1 to 0.4) **0.3 (0.1 to 0.6)** 0.1 (−0.1 to 0.3)
Handgrip strength (kg)	39.1 (11.6) ^1^	**0.5 (0.2 to 0.7)**	**0.5 (0.1 to 0.9)**
Cardiorespiratory fitness (mL/kg/min)	28.3 (7.4) ^2^	**0.6 (0.3 to 0.9)**	0.4 (−0.01 to 0.7)
Functional testing 30STS (n) SLST (s) SCT (s)	15.3 (4.2) ^1^ 48.5 (18.2) ^1^ 8.1 (2.7) ^1^	**1.3 (0.8 to 1.9)** **0.2 (0.1 to 0.4)** **−2.7 (−3.6 to −1.9)**	**0.9 (0.3 to 1.5)** **0.2 (0.02 to 0.3)** **−2.1 (−3.1 to −1.0)**

Multiple linear regression model was used, and the models were adjusted for age, gender, physical work demands, working status (workers vs. non-workers), body mass index, smoking status, number of chronic diseases and hours of leisure-time physical activity. Statistically significant scores are marked in bold (*p* < 0.05). ^1^ missing data from 1 participant; ^2^ missing data from 20 participants.

**Figure A1:**
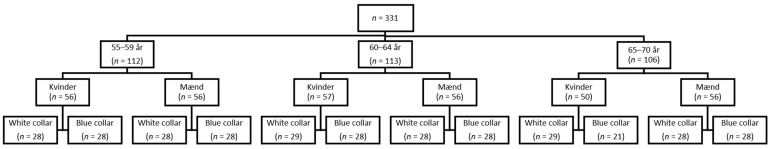
Stratification of clinical substudy. [*n* = numbers].

## Data Availability

The original contributions presented in this study are included in the article. Further inquiries can be directed to the corresponding author.

## Abbreviations

The following abbreviations are used in this manuscript:

WA	Work ability
MCID	Minimal clinically important difference
SD	Standard deviation
STROBE	Strengthening the Reporting of Observational Studies in Epidemiology
WAS	Work ability score
VAS	Visual analog score
LTPA	Leisure-time physical activity
NFA	The National Research Center for the Working Environment
ROM	Range of motion
HGS	Handgrip strength
VO_2_-max	Maximal oxygen uptake
RPM	Revolutions per minute
BPM	Beats per minute
30STS	30 s sit-to-stand
SLST	Single leg stance test
SCT	Stairclimbing test
IPAQ	International Physical Activity Questionnaire Long Form
MET-min/week	Metabolic equivalent minutes per week
BMI	Body mass index
REML	Restricted maximum likelihood estimation
CI	Confidence interval
VIF	Variance inflation factors
*n*	Numbers
IQR	Interquartile range
NA	Not available
KF	Isokinetic knee flexion
SA	Isokinetic shoulder abduction
Nm	Newton meter
Kg	Kilograms
ML	Milliliters
Min	Minutes
n	Repetitions
s	Seconds
REDCap	Research Electronic Data Capture

## Appendix B

**Table A1:** Missing values across all variables in the survey cohort and the clinical substudy. [*n* = numbers; BMI = body mass index; IPAQ = International Physical Activity Questionnaire; NA = not available].

Missing Values	Survey Cohort		Clinical Substudy
	Baseline (*n* = 13,404)	Follow-Up (*n* = 8474)	(*n* = 331)
Age (years), *n* (%)	0 (0%)	0 (0%)	0 (0%)
Gender, *n* (%)	0 (0%)	0 (0%)	0 (0%)
Employment status, *n* (%)	0 (0%)	0 (0%)	0 (0%)
Work ability score (0–100 points), *n* (%)	2870 (21%)	1726 (20%)	0 (0%)
Average weekly working hours, *n* (%)	4253 (32%)	4695 (55%) ^1^	3 (1%)
Self-reported physical work demands, *n* (%)	1355 (10%)	4731 (56%)	0 (0%)
BMI (kg/m^2^), *n* (%)	1430 (11%)	897 (11%) ^2^	0 (0%)
Smoking status, *n* (%)	1262 (9%)	829 (10%)	0 (0%)
Chronic diseases, *n* (%)	1111 (8%)	716 (9%)	0 (0%)
Leisure-time physical activity, *n* (%) Survey questionnaire IPAQ	1291 (10%) NA	829 (10%) NA	NA 26 (10%) ^3^

^1^ three observations dropped due to outliers; ^2^ 11 observations dropped due to outliers; ^3^ 26 observations dropped due to outliers.

## Appendix C

**Table A2:** Baseline differences between responders vs. non-responders at follow-up. [*n* = numbers; SD = standard deviation; BMI = body mass index; LTPA = leisure-time physical activity].

Characteristics	Responders at Follow-Up (*n* = 8474)	Non-Responders at Follow-Up (*n* = 4930)	*p*-Value *
Age (years), mean (SD)	59.1 (4.2)	58.8 (4.3)	**0.001**
Gender, *n* (%) Male Female	4036 (48%) 4438 (52%)	2319 (47%) 2611 (53%)	0.510
Employment status, *n*(%) Regular employment Retired Early retirement Partly subsidized work Unemployed On social security Economic self-supported Other	6146 (72%) 1022 (12%) 695 (8%) 321 (4%) 173 (2%) 55 (1%) 41 (1%) 21 (0%)	3117 (63%) 908 (18%) 358 (7%) 254 (5%) 133 (3%) 109 (2%) 36 (1%) 15 (1%)	**<0.001**
Work ability score (0–100 points), mean (SD)	78.1 (26.5) ^1^	70.7 (31.4) ^2^	**<0.001**
Average weekly working hours, mean (SD)	37.8 (10.3) ^3^	37.6 (11.6) ^4^	0.277
Self-reported physical work demands, *n* (%) High physical work demands Low physical work demands	3750 (47%) ^5^ 4170 (53%)	2340 (57%) ^6^ 1789 (43%)	**<0.001**
BMI (kg/m^2^), mean (SD)	27.0 (4.9) ^7^	27.1 (5.1) ^8^	0.699
Smoking status, *n* (%) Yes Previously smoker Non-smoker	1255 (16%) ^9^ 1752 (22%) 4901 (62%)	1023 (24%) ^10^ 828 (20%) 2383 (56%)	**<0.001**
Chronic diseases, *n* (%) 0 diseases 1 disease 2 diseases 3–4 diseases 5 diseases	5390 (67%) ^11^ 2130 (27%) 425 (5%) 46 (1%) 0 (0%)	2696 (63%) ^12^ 1182 (27%) 353 (8%) 70 (2%) 1 (0%)	**<0.001**
Leisure-time physical activity, *n* (%) None to light LTPA Moderate to high LTPA	4524 (57%) ^13^ 3376 (43%)	2637 (63%) ^14^ 1576 (37%)	**<0.001**

* = *T*-test for continuous variables and chi^2^ test for categorical variables. Statistically significant results (*p* < 0.005) are marked in bold. ^1^ Missing data from 1558 responders, ^2^ missing data from 1312 non-responders, ^3^ missing data from 2293 responders, ^4^ missing data from 1884 non-responders, ^5^ missing data from 554 responders, ^6^ missing data from 801 non-responders, ^7^ missing data from 647 responders, ^8^ missing data from 783 non-responders, ^9^ missing data from 566 responders, ^10^ missing data from 696 non-responders, ^11^ missing data from 483 responders, ^12^ missing data from 628 non-responders, ^13^ missing data from 574 responders, ^14^ missing data from 717 non-responders.

## Appendix D

**Table A3:** Baseline questionnaire differences between participants who participated in the clinical substudy compared to the rest of the cohort. [*n* = numbers; SD = standard deviation; BMI = body mass index; LTPA = leisure-time physical activity].

Characteristics	Clinical Substudy (*n* = 331)	The Rest of the Cohort (*n* = 13,073)	*p*-Value *
Age (years), mean (SD)	57.5 (4.0)	59.0 (4.2)	**<0.001**
Gender, *n* (%) Male Female	168 (51%) 163 (49%)	6886 (53%) 6187 (47%)	0.490
Employment status, *n* (%) Regular employment Retired Early retirement Partly subsidized work Unemployed On social security Economic self-supported Other	244 (74%) 59 (18%) 20 (6%) 1 (0%) 4 (1%) 0 (0%) 3 (1%) 0 (0%)	8932 (68%) 1930 (15%) 1053 (8%) 575 (4%) 306 (3%) 164 (1%) 77 (1%) 36 (0%)	**0.002**
Work ability score (0–100 points), mean (SD) Excellent work ability, *n* (%) Good work ability, *n* (%) Moderate work ability, *n*(%) Poor work ability, *n* (%)	86.1 (16.7) ^1^ 134 (47%) 115 (41%) 22 (8%) 12 (4%)	75.3 (28.7) ^2^ 3811 (37%) 3731 (37%) 837 (8%) 1872 (18%)	**<0.001**
Average weekly working hours, mean (SD)	39.1 (10.5) ^3^	37.7 (10.7) ^4^	**0.022**
Self-reported physical work demands, *n* (%) High physical work demands Low physical work demands	163 (50%) ^5^ 161 (50%)	5927 (50%) ^6^ 5798 (50%)	0.932
BMI (kg/m^2^), mean (SD)	27.3 (4.8)	27.1 (5.0) ^7^	0.323
Smoking status, *n* (%) Yes Previously smoker Non-smoker	33 (11%) ^8^ 62 (20%) 216 (69%)	2245 (19%) ^9^ 2518 (21%) 7068 (60%)	**<0.001**
Chronic diseases, *n*(%) 0 diseases 1 disease 2 diseases 3–4 diseases 5 diseases	242 (77%) ^10^ 62 (20%) 11 (3%) 0 (0%) 0 (0%)	7844 (66%) ^11^ 3250 (27%) 767 (6%) 116 (1%) 1 (0%)	**0.002**
Leisure-time physical activity, *n*(%) None to light LTPA Moderate to high LTPA	161 (52%) ^8^ 150 (48%)	7000 (59%) ^12^ 4802 (41%)	**0.008**

* = *T*-test for continuous variables and chi^2^ test for categorical variables. Statistically significant results (*p* < 0.005) are marked in bold. ^1^ Missing data from 48 participants, ^2^ missing data from 2822 participants, ^3^ missing data from nine participants, ^4^ missing data from 4168 participants, ^5^ missing data from seven participants, ^6^ missing data from 1348 participants, ^7^ missing data from 1408 participants, ^8^ missing data from 20 participants, ^9^ missing data from 1242 participants, ^10^ missing data from 16 participants, ^11^ missing data from 1095 participants, ^12^ missing data from 1271 participants.

## Appendix E

**Table A4:** Analyses from the clinical substudy stratified in workers and non-workers. [*n* = numbers; WAS = work ability score; CI = confidence interval; BMI = body mass index; Nm = newton meter; kg = kilograms; mL = milliliters; min = minutes; 30STS = 30 s sit-to-stand test; SLST = single leg stance test; s = seconds; SCT = stairclimbing test].

Characteristics	Workers (*n* = 245)	Non-Workers (*n* = 86)	*p*-Value *
WAS (0–100 points), mean (95% CI)	83.8 (81.5 to 86.0)	64.7 (58.5 to 70.9)	**<0.001**
Age (years), mean (95% CI)	60.9 (60.4 to 61.4)	65.5 (64.9 to 66.2)	**<0.001**
Gender, *n* (%) Males Females	131 (53%) 114 (47%)	37 (43%) 49 (57%)	0.096
Physical work demands, *n* (%) High physical work demands Low physical work demands	114 (47%) 131 (53%)	40 (47%) 46 (53%)	0.998
BMI (kg/m^2^), mean (95% CI)	27.3 (26.7 to 27.9)	27.5 (26.5 to 28.6)	0.646
Knee (Nm), mean (95% CI) Isometric extension Isokinetic extension Isokinetic flexion	170.8 (163.1 to 178.5) ^1^ 132.9 (128.0 to 137.9) 68.6 (65.9 to 71.3)	153.7 (142.9 to 164.4) 120.8 (113.9 to 127.7) 59.2 (55.2 to 63.2)	**0.016** **0.010** **>0.001**
Shoulder (Nm), mean (95% CI) Isometric abduction Isokinetic abduction Isokinetic adduction	53.4 (51.2 to 55.7) 47.7 (45.8 to 49.7) 70.1 (66.8 to 73.3)	49.0 (45.2 to 52.8) ^1^ 44.3 (41.2 to 47.5) 60.8 (55.7 to 65.9)	0.052 0.081 **0.004**
Handgrip strength (kg), mean (95% CI)	40.0 (38.6 to 41.5) ^1^	36.6 (34.2 to 38.9)	**0.0173**
Cardiorespiratory fitness (mL/kg/min), mean (95% CI)	29.0 (28.0 to 30.0) ^2^	26.2 (24.8 to 27.6) ^3^	**0.003**
Functional testing, mean (95% CI) 30STS (n) SLST (s) SCT (s)	15.8 (15.2 to 16.3) ^1^ 49.8 (47.6 to 52.0) ^1^ 7.8 (7.4 to 8.1) ^1^	14.0 (13.2 to 14.8) 44.7 (40.3 to 49.0) 9.2 (8.6 to 9.8)	**0.001** **0.024** **<0.001**

* = *t* test for continuous variables and chi^2^ for categorical variables. Statistically significant scores are marked in bold (*p* < 0.05). ^1^ missing data from one participant, ^2^ missing data from 14 participants, ^3^ missing data from six participants.
